# Research Progress and Prospects of Molecular Marker Technology on Jujube Trees

**DOI:** 10.3390/plants15182789

**Published:** 2026-09-11

**Authors:** Yanxu Liu, Ruijia Li, Zhihui Zhao, Mengjun Liu, Lili Wang

**Affiliations:** 1 College of Horticulture, Hebei Agricultural University, Baoding 071001, China; 17535156073@163.com (Y.L.);; 2 Research Center of Chinese Jujube, Hebei Agricultural University, Baoding 071001, China

**Keywords:** jujube, molecular markers, germplasm resources, genetic relationship, genetic diversity, gene mapping, genetic map

## Abstract

Jujube is an important fruit tree and one of the five major economic forest tree species native to China, possessing high nutritional and medicinal value. It plays a key supporting role in the efficient utilization of marginal land resources—such as mountainous, sandy, saline-alkali, and drought-prone areas—as well as in the revitalization of rural industries. Molecular marker technology, with its advantages of stability, accuracy, and efficiency, has become an important tool in jujube genetic breeding research and is widely applied. This article summarizes molecular markers based on three generations of technological intergenerational systems: the first generation of hybridization-based markers (Restriction Fragment Length Polymorphism, RFLP), the second generation of PCR-based markers (represented by Simple Sequence Repeat, SSR), and the third generation of high-throughput sequencing markers (Single Nucleotide Polymorphism, SNP, Genotyping-by-Sequencing, GBS, etc.). A comprehensive classification system based on technical principles, polymorphism sources, and other dimensions is constructed to clearly explain the driving forces and development trends of molecular marker system evolution in jujube tree research, and to clarify the selection criteria and adaptation strategies of molecular markers. Research has found that the second-generation molecular marker SSR is the fundamental core tool for standardized identification of jujube germplasm resources and genetic analysis of low-budget populations. The third-generation molecular markers represented by SNPs and InDel are high-density maps, Genome-Wide Association Study(GWAS), Genomic selection, and other modern precision breeding core carriers, forming a layered complementary technology system, and are gradually becoming the mainstream of research. This paper summarizes the application progress of molecular markers in the precise identification of jujube germplasm resources, analysis of genetic diversity, determination of genetic relationships, construction of high-density genetic maps, genome-wide association analysis, functional gene mapping, and tracing of domestication and evolution. This article integrates all existing research using the unified scientific framework of “technology defect driven tagging iteration”, analyzes the internal logic of the evolution and replacement of different tagging systems, the inherent limitations of early tagging, the existing problems in current research, and discusses future research directions, aiming to provide a reference for the scientific and efficient application of molecular markers in jujube and to promote the improvement and upgrading of the jujube molecular marker-assisted breeding technology system.

## 1. Introduction

### 1.1. Value and Research Significance of Jujube

Jujube (*Ziziphus jujuba* Mill.) belongs to the genus Ziziphus of the family Rhamnaceae and is derived from the wild species Ziziphus acerba [1]. It is a unique and economically important fruit tree and one of the five major economic forest tree species in China, with a long history of cultivation [2]. Jujube trees exhibit outstanding biological characteristics, including drought resistance, salt and alkali tolerance, and adaptability to poor soils [3], making them ideal tree species for both ecological restoration and income generation in mountainous, sandy, saline-alkali, and drought-prone areas. The fruits are sweet and can be consumed fresh or dried. They are nutritionally rich and possess medicinal effects such as tonifying qi (vital energy), nourishing blood, protecting the liver, strengthening the spleen, and beautifying the skin [4], rendering them highly favored by consumers. Therefore, jujube trees hold significant economic, ecological, and social value in the development and utilization of marginal lands, increasing farmers’ income, revitalizing rural industries, and ensuring national health. Conducting research on jujube genetic breeding is of great importance for promoting the high-quality development of the jujube industry.

### 1.2. Development and Research Application of Molecular Marker Technology

Molecular markers refer to genetic polymorphism markers acquired via specific technical approaches based on DNA-level genetic variations. In comparison with traditional identification methods, including morphological, cytological, and palynological methods, molecular markers are not influenced by environmental conditions, sample types, or sampling periods. They possess the advantages of high stability, high accuracy, rapid detection, and convenient operation. Consequently, they have emerged as core technologies in the domains of plant trait genetic analysis, molecular breeding, and biotechnology research.

#### Definition of Core Terminology Related to Molecular Breeding

Molecular breeding refers to a complete breeding technology system that relies on genetic variations in genomic DNA to evaluate germplasm resources, locate target genes, and improve economic traits. It includes two core technical branches: marker-assisted selection and genomic selection. Marker-assisted selection (MAS) refers to the use of a small number of molecular markers linked to the main quality trait to screen hybrid offspring, which is suitable for improving single-gene control traits such as disease resistance and skin color. Marker-assisted backcrossing (MABC) is a method that combines foreground markers to screen for excellent target genes and background markers to quickly restore the genetic background of recurrent parents, and is used for targeted improvement of existing cultivated varieties. Genomic selection (GS) is a statistical prediction model constructed based on high-density Single Nucleotide Polymorphisms (SNPs) throughout the genome, which estimates the comprehensive breeding value of multiple individual traits at once and adapts to multiple genes controlling quantitative traits such as fruit size and sugar content. Genome prediction is the core computational process of genome selection, which predicts the phenotypic potential and breeding value of unknown materials through individual genotype data.

Molecular marker technology has experienced three generations of development. The first generation, typified by RFLP and founded on Southern hybridization technology, has been gradually phased out. Despite its high stability, its complex operation has led to this outcome. The second generation, including Simple Sequence Repeat (SSR), Amplified Fragment Length Polymorphism (AFLP), and Inter-simple sequence repeat (ISSR), which is based on PCR amplification technology, has been extensively utilized owing to its simplicity and abundant polymorphism. The third generation, such as SNP, Insertion‑Deletion (InDel), Restriction site-associated DNA (RAD), and Genotyping-by-Sequencing (GBS), relying on high-throughput sequencing technology, integrates high-throughput and high precision, and has emerged as the mainstream technology in contemporary plant molecular genetic research. With the advancement of high-throughput sequencing technology and the reduction in detection costs, the development and application of molecular marker technology have been continuously enhanced, offering robust technical support for the evaluation of plant germplasm resources, the construction of genetic maps, the exploration of functional genes, and molecular design breeding.

### 1.3. Progress and Current Situation of Molecular Marker Research in Jujube

Research on molecular markers in jujube trees commenced relatively late, and there were no reports on the application of the first-generation molecular markers founded on Southern hybridization technology. The second-generation molecular markers based on PCR technology signified the onset of molecular marker research in jujube trees. As early as 1992, our research team initiated the utilization of Random Amplified Polymorphic DNA (RAPD) markers to identify different variants of golden-threaded small jujube and seedless jujube and conduct an analysis of their genetic relationships [5], thereby pioneering the research on molecular markers in jujube trees. Subsequently, second-generation molecular markers, such as AFLP, SSR, and ISSR, were successively employed in the identification of jujube tree varieties, the analysis of genetic diversity, and the exploration of genetic relationships. In particular, the development of efficient and universal SSR primers for jujube at the whole-genome level [6,7] and the establishment of SSR identification standards for jujube tree varieties [8] facilitated the standardized application of second-generation molecular markers in jujube tree research.

The utilization of third-generation molecular markers founded on high-throughput sequencing technology in jujube tree research commenced relatively early. Our research group successfully established a high-density molecular genetic linkage map by employing SNP and RAD markers during the initial whole-genome sequencing and assembly of jujube trees [9], which provided the groundwork for the application of third-generation molecular markers. At present, among the second-generation molecular markers, only SSR remains commonly utilized in jujube tree research. In contrast, third-generation molecular markers such as SNP and InDel are gradually emerging as the mainstream and are extensively applied in the construction of high-density genetic maps, mapping of functional genes, analysis of important traits, as well as research on domestication and evolution of jujube trees. Simultaneously, the publication of high-quality jujube genomes, particularly the (T2T) haplotype genomes [10,11], has laid a robust genomic basis for the development and efficient utilization of molecular markers in jujube trees, propelling the research on molecular markers in jujube trees into a new phase of high-throughput and precision. In recent years, breakthroughs have been made in the study of jujube pan genome, systematically analyzing the Presence‑Absence Variation (PAV), copy number variation (CNV), and large fragment structural variation (SV) of the whole genome. The above structural variations are the core genetic variations that regulate fruit quality and stress resistance in fruit trees. Traditional SSRs and single nucleotide SNPs can only capture a small amount of variation and cannot cover all trait regulatory sites. Multi-level genomic resources jointly support the research of molecular markers in jujube trees to enter a new stage of high-throughput and precision.

## 2. Classification, Characteristics, and Selection Strategies of Molecular Markers

### 2.1. Classification System of Molecular Markers

Molecular markers serve as crucial instruments for tracing genetic variations within species and pinpointing specific functional genes. Plant molecular markers can be systematically categorized according to diverse dimensions, including technical principles, sources of polymorphism, genetic characteristics, and functional correlations. Each type of marker exhibits distinct technical traits and appropriate application scenarios.

#### 2.1.1. Classification by Technical Principles

First-generation molecular markers: Rooted in Southern hybridization technology, these markers were initially put forward in 1980 [12], with RFLP serving as the representative. This marker identifies differences in fragment lengths by cleaving genomic DNA with restriction endonucleases and integrating with probe hybridization technology. It features codominance and high stability; however, the operation process is intricate, requiring known probes and large DNA quantities. As a result, it has largely been phased out from plant research applications.

Second-generation molecular markers: Based on PCR amplification technology, they emerged in 1990 [13] and can be further classified into three categories. The first category consists of markers based on random primer PCR amplification, RAPD and (ISSR) markers. RAPD exhibits poor repeatability and is seldom utilized at present, whereas ISSR demonstrates a higher level of polymorphism and better stability compared to RAPD. The second category comprises markers based on specific primer PCR amplification, including microsatellites (SSR), sequence-tagged sites Sequence Tagged Site (STS), and sequence-characterized amplified regions Sequence-characterized amplified region (SCAR) markers. Among them, SSR markers possess strong stability and abundant polymorphism and are currently commonly employed in plant research [14], while the application scope of STS and SCAR markers is relatively restricted. The third category is markers based on the combination of PCR and enzyme digestion, represented by amplified fragment length polymorphism (AFLP), which has rich polymorphism and is suitable for the study of species lacking genomic sequence information.

Third-generation molecular markers: Based on high-throughput sequencing technology, SNP large-scale typing technology has gradually developed and matured since the mid to late 1990s [15]. With the popularization of second-generation sequencing, it has become the mainstream molecular marker of the third generation, which can capture single-base variations in the entire genome on a large scale, mainly including four types. Single nucleotide polymorphism (SNP) markers, which detect single-base differences in the genome, are widely distributed, have rich polymorphism, and have achieved high-throughput and low-cost detection. Insertion/Deletion (InDel) markers [16], developed based on short fragment insertions or deletions in the genome, are straightforward to detect and can be genotyped in high-throughput. Restriction site-associated DNA (RAD) and genotyping-by-sequencing (GBS) markers belong to simplified genome sequencing technology. Having a standardized typing process: genomic DNA digestion → linker ligation → library construction → high-throughput sequencing → alignment with reference genome → SNP genotyping. RAD recommends sequencing depths of 10–20×, suitable for constructing medium density genetic maps. GBS recommends sequencing at a depth of 5–10×, which is lower in cost. However, low-depth sequencing can generate a large number of missing genotypes, which need to be filled in through bioinformatics algorithms. Otherwise, it will significantly reduce the accuracy of linkage disequilibrium calculation and GWAS localization. High-throughput SNP genotyping markers based on chip technology (e.g., KASP markers) exhibit high reproducibility and automated operation, making them suitable for large-scale population genetics studies involving hundreds to thousands of germplasm samples. These markers can reduce false-positive associations in GWAS through permutation tests and population structure correction.

#### 2.1.2. Classification by Polymorphism Source

In accordance with the fundamental source of genetic polymorphism, molecular markers can be classified into four categories: Markers predicated on disparities in DNA restriction fragment lengths (e.g., RFLP, AFLP). Markers based on variations in the lengths of genomic repetitive sequences (e.g., SSR, ISSR). Markers based on single-nucleotide base variations (SNP). Markers based on insertions or deletions of short genomic sequence fragments (InDel).

#### 2.1.3. Classification by Genetic Characteristics

Based on the genetic expression characteristics of the markers, they can be classified into dominant markers and codominant markers. Dominant markers are unable to differentiate between homozygous and heterozygous genotypes, such as RAPD and AFLP. Codominant markers can precisely distinguish between homozygous and heterozygous genotypes, with a more comprehensive expression of genetic information, such as RFLP, SSR, SNP, etc.

#### 2.1.4. Classification by Functionality

Based on the degree of association between the markers and plant functional genes, they can be classified into neutral markers and functional markers. Neutral markers only represent genetic loci on the genome and do not directly participate in the regulation of target traits. Functional markers are derived from sequence variations in the coding or regulatory regions of trait-related genes, theoretically directly linked to phenotypes.

A diversity of genic-associated molecular markers have been widely exploited in fruit-tree genetic studies. Anonymous SSRs originate from random non-coding genomic repetitive sequences and represent typical neutral markers that detect length polymorphisms of simple-sequence repeats. By contrast, gene-derived SSRs are developed from sequences of known or predicted candidate genes and hold greater potential to be converted into functional markers compared with anonymous SSRs, among which Expressed Sequence Tag-Simple Sequence Repeats (EST-SSRs) constitute a specific subclass mined from transcriptome assemblies of transcribed gene regions. Cleaved Amplified Polymorphic Sequence (CAPS) markers rely on naturally occurring restriction-enzyme recognition sites created or abolished by target-locus single-nucleotide polymorphisms, whereas Derived Cleaved Amplified Polymorphic Sequence (dCAPS) circumvents the requirement for native restriction sites by introducing artificial mismatches in PCR primers to generate novel cleavage sites for genotyping. Compared with conventional SSRs, CAPS and dCAPS anchor to specific genic sequences and are better suited for candidate-gene validation; nevertheless, they demand higher labor input for single-marker development and exhibit markedly lower throughput than high-density SNP genotyping, making them unsuitable for genome-wide genetic-diversity surveys. Functional SNPs are sequence variants residing within coding or regulatory gene regions that may alter protein structure or gene expression and potentially constitute causal variants for phenotypic traits. It should be emphasized that KASP (Kompetitive Allele-Specific PCR) is not a standalone molecular-marker type but a high-throughput genotyping assay platform that can genotype pre-defined neutral or functional SNP loci using allele-specific fluorescent primers. The category, advantages, and limitations of these markers and assay systems are summarized in Table 1.

Neutral markers have no direct association with plant functional genes and merely serve as markers for specific genetic loci in the genome, such as RFLP, RAPD, AFLP, SSR, ISSR, etc. Functional markers are developed based on the sequences of functional genes related to plant phenotypic traits, with marker sites located in the coding or regulatory regions of functional genes, and are directly associated with phenotypic traits, such as SNP and EST-SSR markers related to disease resistance, yield, quality, etc.

#### 2.1.5. Other Special Types

Apart from the aforementioned mainstream classifications, there are also certain special types of molecular markers that integrate multiple technologies. Cleaved Amplified Polymorphic Sequence (CAPS) markers combine specific primer PCR amplification and restriction enzyme digestion technology to detect polymorphisms through the digestion of PCR products. Expression Sequence Tag (EST) markers are developed based on plant cDNA expression sequence tags, integrating both gene expression information and genetic polymorphism, and are frequently employed for functional gene mining and mapping.

### 2.2. Comparison of Technical Characteristics of Common Molecular Markers

Distinct types of molecular markers exhibit substantial disparities in genetic traits, polymorphism, reproducibility, sequence prerequisites, and technical intricacy. Moreover, their detection throughput and application expenses also differ, which directly determine the appropriate application scenarios for these markers. The core technical characteristics of common molecular markers are presented in a comparative manner in Table 2.

Overall, molecular markers such as (RFLP), AFLP, RAPD, and SCAR have gradually phased out from the research and application of jujube trees due to technical drawbacks or application limitations. At present, the most extensively employed markers in plant research are Simple Sequence Repeat (SSR) and Single Nucleotide Polymorphism (SNP) markers. SSR markers feature moderate costs. Although they suffer from the disadvantages of time-consuming primer design and strong species specificity, these issues have been effectively addressed with the popularization of species genome sequencing. SNP markers exhibit high detection throughput and low unit data cost, yet the initial investment in chips or sequencing platforms is relatively substantial. In practical research, the technical advantages of diverse types of molecular markers can be comprehensively harnessed to achieve complementary strengths.

Based on the genomic characteristics of jujube trees (high heterozygosity, small genome size), further horizontal comparison of mainstream marker systems is conducted in terms of polymorphic information content (PIC), experimental repeatability, detection cost, detection flux, and difficulty in automation implementation. SSR markers have high polymorphic information content and were widely used for jujube germplasm evaluation in the early stages, but automated typing is difficult to support genome-wide association analysis. Third-generation markers such as SNPs and GBS have a high degree of automation and huge detection throughput, making them more suitable for genome-wide association analysis (GWAS) and genome selection (GS). The above genomic features directly explain the large-scale use of SSR in early research on jujube trees, and the inherent reason for the current shift towards SNP typing technology at the whole genome level.

In the modern genome precision breeding system, SSR is only suitable for low-cost germplasm pre-screening, small-scale germplasm fingerprinting construction, and rapid identification of variety rights. Its inherent weakness is the extremely low density of whole-genome coverage, which makes it impossible to capture multiple genes with small effects, and is not compatible with automated high-throughput typing, whole-genome association analysis (GWAS), and whole-genome selection (GS). SNP/InDel, with its advantages of ultra-high whole-genome marker density, uniform coverage, automated batch typing, native adaptation to GWAS, and genome selection, has become the core carrier for precise genetic analysis and molecular design breeding of modern jujube trees. There is no technological substitution relationship between the two, and it is recommended to use a hierarchical combination: SSR completes the primary typing of germplasm, and SNP carries out high-density gene mapping and complex trait genetic analysis. The cost of SSR markers is moderate. Although there are drawbacks such as time-consuming primer design and strong species specificity, with the popularization of jujube genome sequencing data, the efficiency of primer development has significantly improved. The cost of SNP single-sample unit locus detection is lower, but the initial equipment investment for second-generation sequencing and SNP chip platforms is higher. In practical research, the technological advantages of different types of molecular markers can be comprehensively utilized to achieve complementary advantages.

### 2.3. Selection Basis and Adaptation Strategies for Molecular Markers

#### 2.3.1. Selection Basis for Molecular Markers

The selection and application of molecular markers require comprehensive consideration of four core factors. Priority should be given to choosing marker types that are suitable for research needs, rather than blindly pursuing new and high-cost marker technologies. The first factor is the research purpose. Different research directions have different requirements regarding marker polymorphism, throughput, and genetic characteristics, which serve as the core basis for marker selection. The second is the technical characteristics of the markers. It is necessary to select markers that match the research conditions based on their genetic characteristics, polymorphism, repeatability, and technical complexity, as shown in Table 1. The third is the characteristics of the research species, including genome complexity, ploidy level, and existing genetic information, which directly determine marker applicability. The fourth is technical feasibility and cost-effectiveness. It is necessary to balance detection efficiency and cost, taking into account the laboratory’s hardware conditions, researchers’ technical experience, and available research funds. With the rapid development of high-throughput sequencing technology and the continuous reduction in detection costs, high-throughput markers (e.g., SNP and GBS) and functional markers are being increasingly widely used, becoming core technologies for advancing precision plant breeding, molecular design breeding, and domestication and evolution research.

#### 2.3.2. Adaptation Selection Strategies for Molecular Markers

In studies of genetic diversity analysis, phylogenetic relationship inference and complex genetic trait dissection, molecular markers characterized by high polymorphism and broad genomic coverage (e.g., SSR, SNP, and AFLP) are preferentially selected [17]. For cultivar authentication and DNA fingerprinting construction, SSR markers featuring simple operation, low experimental cost, and high genotyping stability are recommended. For genetic map construction, gene mapping, and QTL analysis, high-density codominant markers such as SNP, SSR, and AFLP are required [18]. For marker-assisted breeding programs, markers that are tightly linked to target agronomic traits, including SSR, SNP, and KASP, should be given priority. For the cloning and functional mining of plant functional genes, gene expression-associated markers such as EST and SNP are more suitable for application. In summary, the selection of molecular marker types must be strictly aligned with specific research objectives, as different research tasks require the adoption of corresponding suitable molecular marker types.

Depending on the genomic and genetic characteristics of the target species, codominant markers including SSR and SNP are required when distinguishing between homozygous and heterozygous genotypes. For species with simple genome composition, the application of SNP and InDel markers can effectively improve genotyping detection efficiency. For species with complex genomes, SSR and AFLP markers can deliver higher genotyping stability. For species with available complete genetic information, priority should be given to previously published markers or SSR/SNP markers developed based on existing reference genomes. For species without available whole genomic sequence data, genome sequence-independent molecular markers such as RFLP, AFLP, and ISSR are the preferred selection. For polyploid species, markers that can effectively distinguish homoeologous genes, such as SSR, are more suitable. Overall, the selection of molecular markers must be based on the target species’ genomic structure, ploidy level, and the availability of existing genetic resources.

When experimental conditions are taken into consideration, for research with small sample sizes, high-throughput markers that require only low DNA input—represented by KASP [19]—are the optimal choice. For research with high throughput requirements, high-throughput markers such as SNP and RAD can be employed. When constrained by research budgets, low-cost markers including SSR and ISSR are feasible selection options. For the rapid screening and evaluation of germplasm resources, fluorescently labeled SSR or KASP markers are more suitable. In summary, the selection of molecular markers should be flexibly adjusted according to the laboratory’s existing equipment configuration, research funding allocation, and sample size.

Furthermore, the compatibility of candidate markers with downstream bioinformatic data analysis should also be incorporated into the consideration of marker selection. Markers that have been functionally validated and confirmed to have stable amplification performance in the target species or its closely related species should be given priority. For multi-institutional collaborative research projects, the consistency and comparability of genotyping data generated across different experimental platforms and laboratories must be strictly guaranteed. Pre-experimental validation of candidate markers is an essential step, which can be used to optimize PCR amplification conditions and confirm the polymorphism, repeatability, stability, and applicability of the selected markers before formal experiments are carried out. To sum up, all marker selection strategies need to be verified through preliminary experiments to ensure the quality of obtained genotyping data and the reliability of final experimental results.

#### 2.3.3. Horizontal Comparison of Molecular Marker Selection for Perennial Fruit Trees

Compare industrialized mature perennial fruit trees such as apples, pears, peaches, and grapes. The development of grape SSR markers started earliest [20] and has established a globally recognized standard for germplasm fingerprint identification. Apple and peach have completed commercial high-density SNP typing chips [21,22] and standardized trait-linked KASP functional marker panels, which are widely used in whole-genome selection breeding.

There is a significant gap between jujube trees and mainstream fruit tree molecular breeding technologies: Standardized commercial SNP chips have not yet been developed, triploid germplasm has a high proportion, suitable SNP typing algorithms are missing, and a large-scale genome selection system has not been established. The molecular marker system for jujube trees can focus on drawing on the mature technology paths of peach and apple, such as constructing dedicated SNP chips, developing a complete set of KASP functional markers, and building a multi-environment genome selection prediction model to shorten the gap with mainstream fruit tree molecular breeding technologies.

## 3. Progress in the Application of Molecular Marker Technology in Jujube Trees

### 3.1. Application of Second-Generation Molecular Markers in Jujube Research

**Figure 1:**
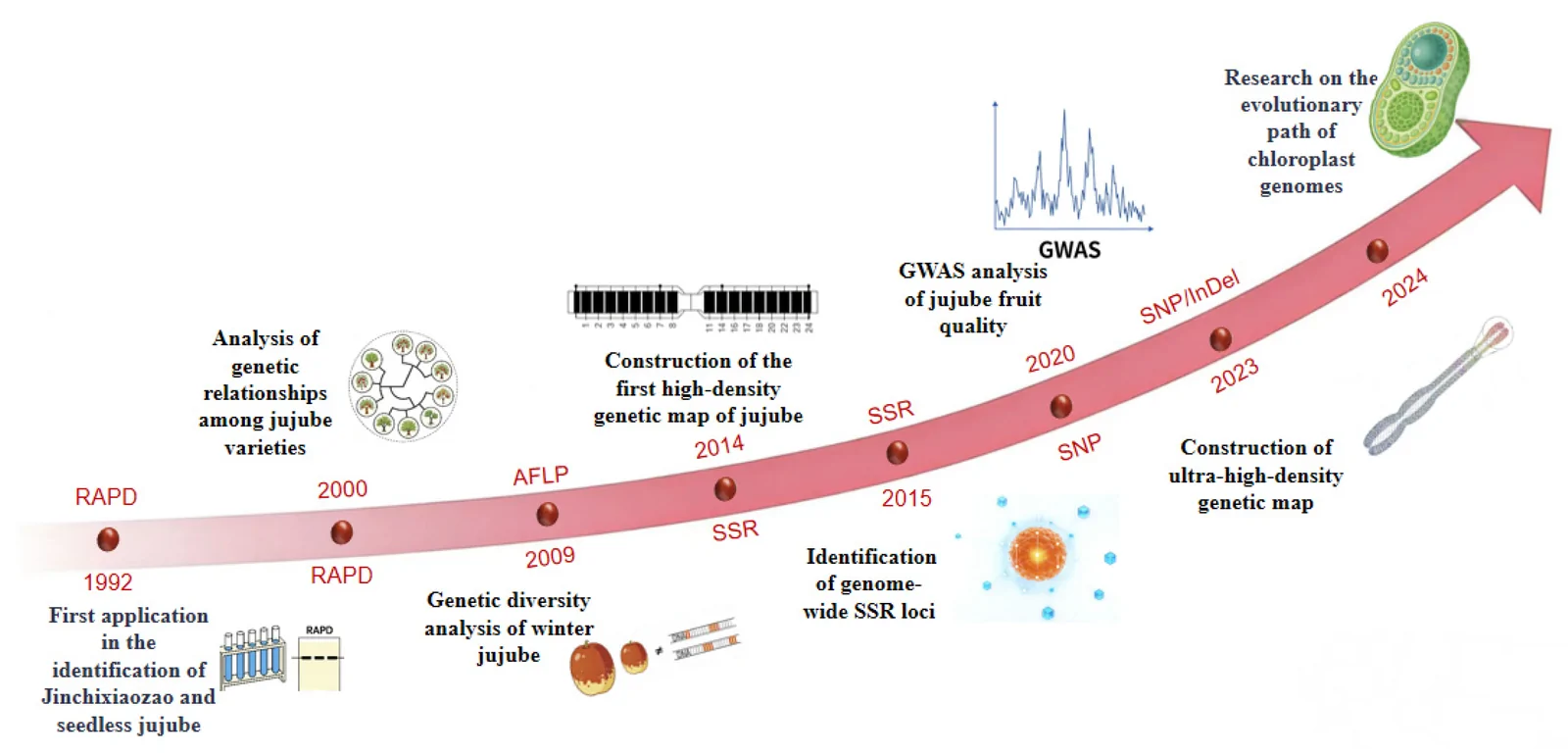
Development trend of molecular marker applications in jujube trees over time.

The emergence of second-generation molecular markers based on PCR amplification technology marked the beginning of jujube molecular marker research. Markers such as RAPD, AFLP, ISSR, and SSR were successively applied in jujube studies. Among them, SSR markers, due to their strong stability and rich polymorphism, became the core markers of this generation, promoting research progress in jujube germplasm resource evaluation, genetic relationship analysis, and early genetic map construction (Figure 1).

#### 3.1.1. Germplasm Resource Identification and Genetic Relationship Analysis

The phenomena of “same name but different species” and “same species but different names” are widespread in jujube germplasm resources [23,24]. Traditional morphological and cytological markers are insufficient for accurate differentiation. Second-generation molecular markers have become important tools for the precise identification of jujube germplasm resources and the determination of genetic relationships.

Early RAPD markers were only suitable for rough differentiation of germplasm. Ye Chunxiu et al. [25] used RAPD technology to accurately distinguish two closely related varieties—Hami Yuzao and Hami Dazao—in Xinjiang using only two primers. Peng Jianying et al. [26] and Liu Dongzhi et al. [27] used this marker to analyze the genetic distance of jujube, sour jujube, and dragon jujube varieties. Random amplified polymorphic DNA (RAPD) is a dominant marker that cannot distinguish between homozygous and heterozygous genotypes. Its amplification repeatability is poor, making it difficult to establish a standardized germplasm identification database. Currently, it has been largely withdrawn from jujube tree research.

In the medium term, AFLP and ISSR are primarily used for regional germplasm differentiation assessment. Ahmad, Riaz [28] successfully distinguished phenotypically similar genotypes through ISSR. Abdel Satter, Mahmoud [29] carried out molecular marker studies employing ISSR markers to distinguish species and identify the relationships among Indian jujube varieties. The findings indicated that there were disparities in various morphological indicators and color values among different varieties. Moreover, there are differences in genetic diversity parameters associated with the relationship between matK barcodes, ISSR markers, and Indian jujube varieties. Through cluster analysis, it was discovered that the prickly *‘Zytoni’* and *‘Um Sulaem’* appear to be distinct single branches from other varieties, which is related to differences in gene expression levels. The experimental operation of AFLP markers and ISSR markers is cumbersome, with no universal standardized primers, and cross-laboratory data cannot be horizontally compared. Only a small number of regional small-scale germplasm studies are used.

SSR markers are the standardized core tool of second-generation markers, which solves the shortcomings of poor repeatability and unstable typing in the first two types of markers. Shang Guoli et al. [30] combined SSR molecular markers with flow cytometry to identify that all 18 hybrid progenies of ‘Dongzao’ and ‘Chenguang’ were triploids. Li Bin et al. [31] used MGB-SSR methods to screen out 12 pairs of polymorphic SSR primers, successfully distinguishing 249 jujube genotypes and achieving precise identification of triploid jujube genotypes. Fu Pengcheng [32] developed 17 pairs of validated simple sequence repeat (SSR) primers that are suitable for the identification of jujube germplasm resources. Liu Xiuyun et al. [33] used six pairs of SSR primers to analyze 39 samples of cultivated jujube, wild jujube, and hairy-leaf jujube germplasm, finding that cultivated jujube and wild jujube had a relatively close genetic relationship, whereas hairy-leaf jujube exhibited a greater genetic distance from both. Bao Wenhui et al. [34] used 19 pairs of SSR primers to confirm that there was a close genetic association among jujube varieties in Beijing. Li Hui et al. [35] used SSR markers to discover that jujube varieties within the same geographical area showed significant genetic correlations. However, SSR markers have extremely low genome-wide marker coverage density and can only capture a small number of major effect sites, making it difficult to analyze the micro effect multi genes that control fruit quality and stress resistance. This makes it difficult to support precise mapping of complex quantitative traits and whole-genome selection breeding. In addition, Li Li et al. [36] first applied SRAP markers to the study of genetic relationships within the genus Ziziphus, confirming that the genetic similarity coefficient could effectively reflect the genetic relationships among species in this genus.

Overall, there are still several prominent practical problems in the application of second-generation markers: The lack of a standardized SSR core marker panel across different jujube germplasm resource libraries. Partial SSR markers have limited transferability between wild jujube and cultivated jujube materials. Due to the small size of hybrid populations and interference from genotype–environment interactions, it is difficult to convert marker trait associations into stable and reusable QTL loci.

#### 3.1.2. Genetic Diversity Analysis and Core Germplasm Construction

SSR, AFLP, and ISSR are the main markers for evaluating the genetic diversity of jujube trees. All similar studies can be summarized as follows: SSR analysis accuracy is significantly higher than dominant markers, and it can fully capture the allelic diversity of the population. Wu Cheng [37], Zhang Zhendong [38], Chiou Chuying et al. [39], Wang Siqi et al. [40], and Pang Xiaoming et al. [41] confirmed the rich genetic diversity of Chinese jujube and Indian jujube germplasm through SSR and constructed 150 core germplasm banks based on 962 materials. Xiao Jing et al. [42] compared fruit trees and found that the abundance of SSR genes in jujube trees was higher than that in apples, pears, and grapes. Liang Tian et al. [43] developed nuclear SSR markers to analyze the germplasm differentiation of multi-country hairy jujube. AFLP and ISSR are commonly used for regional small-scale germplasm diversity assessment (Reza Shahhoseini [44], Singh, S. K [45], Shen Jie et al. [46]), which can only obtain the overall differentiation trend of the population and cannot finely analyze the population structure and linkage disequilibrium patterns.

In the assessment of genetic diversity, in addition to the polymorphism of the markers themselves, the transferability of markers and the absence of a unified core marker set also constrain the horizontal comparison of data between different studies. The existing SSR primer sets used in different studies are inconsistent, making it difficult to directly compare the genetic diversity results of different germplasm banks.

#### 3.1.3. Preliminary Construction of Early Genetic Maps

The second-generation molecular markers can only construct low-density linkage maps, with large marker intervals and insufficient saturation of linkage groups. They can only roughly locate major genes and cannot resolve quantitative trait loci. They only serve as the preliminary basis for the third-generation high-density maps.

### 3.2. Application of Third-Generation Molecular Markers in Jujube Research

The third-generation molecular markers founded on high-throughput sequencing technology, such as single nucleotide polymorphism (SNP), insertion-deletion (InDel), restriction site-associated DNA sequencing (RAD), and genotyping-by-sequencing (GBS), possess the features of high throughput, high precision, and wide genomic coverage. These markers are appropriate for in-depth research requirements, including the establishment of high-density genetic linkage maps, the localization of genes related to important traits, and the genomic analysis of domestication and evolution in jujube. However, there are inherent technical defects in the sequencing and typing processes, which have gradually become the mainstream of molecular marker research in jujube trees, propelling jujube molecular genetic research into a high-throughput and precise phase.

#### 3.2.1. Construction and Optimization of High-Density Genetic Linkage Maps

Important economic traits of jujube, such as fruit size, quality, and stress resistance, are all complex quantitative traits. The analysis of their genetic basis requires the support of high-density genetic maps. Third-generation molecular markers have become the core technology for constructing high-density genetic linkage maps in jujube, and the density and saturation of these maps have been continuously improved. There are limitations in differential technology for whole-genome resequencing typing, and low-depth GBS (<5×) can produce a large number of missing genotypes, directly reducing the density of map markers. The decay rate of linkage disequilibrium (LD) in jujube trees is moderate, and only high-density SNP markers can meet the fine QTL mapping threshold. Zhao Jin et al. [9] used RAD to simplify genome sequencing and construct the first high-density map of jujube. Tang Haixia et al. [47] constructed a hybrid population map based on GBS-SNP. Guo Tianfa et al. [48] and Yan Fenfen et al. [49] obtained tens of thousands of SNP/InDel markers through whole-genome resequencing, and built the currently most saturated jujube genetic map with an average marker interval of only 0.2 cm. However, this technology also has limitations, relying on high-quality T2T reference genomes. If there are duplicate region gaps in genome assembly, false positive SNP variations may occur. The SNP typing algorithm for triploid jujube germplasm is not yet mature, and the error in identifying heterozygous loci is relatively high.

#### 3.2.2. Gene Mapping and Mining of Important Traits

Third-generation molecular markers can precisely reflect genomic genetic variations. When combined with genome-wide association analysis (GWAS) and QTL-seq technology, they enable accurate mapping and mining of genes related to important traits in jujube, thereby providing target genes for jujube molecular breeding. GWAS combined with SNP markers to mine loci associated with drought resistance, fruit quality, and domestication traits in jujube trees. Li Jingzu et al. [50] and Guo Mingxin et al. [51] located a large number of trait-associated SNPs in 150–1059 germplasms of different sizes; Pan Yiling et al. [52] used QTL seq to screen candidate genes for fruit size.

However, from a methodological perspective, the existing jujube tree GWAS system has obvious shortcomings, mainly in the following aspects. Firstly, it can only detect major effect sites with high effect values, and cannot identify minor effect genes that regulate quality and stress resistance. Secondly, the vast majority of domestic jujube tree GWAS research only uses a single mixed linear model (MLMM), which only corrects the basic population structure. Multi-site GWAS models such as FarmCPU and BLINK can effectively reduce false positive associations and improve the detection rate of micro effect sites, but their application in jujube tree research is very limited and represents a clear methodological gap. Thirdly, most studies have not systematically conducted PCA population stratification correction, kinship matrix construction, missing data filling, and multiple test threshold correction, which can easily generate a large number of false positive loci without breeding value. Fourthly, the existing GWAS are all single-environment and single-trait analyses, lacking a multi-environment and multi-trait composite GWAS system, which cannot analyze the regulatory mechanism of genotype–environment interaction on economic traits of jujube trees.

In addition, relying solely on GWAS makes it difficult to accurately screen functional candidate genes. The current mainstream solution is multi-omics joint analysis. Integrating RNA seq transcriptome differentially expressed genes, expression quantitative trait loci (eQTL), and gene co-expression networks can quickly narrow down the candidate gene range within the association interval. Metabolomics and proteomics can verify the regulatory functions of variations on fruit flavor and resistance pathways. However, there is a scarcity of research cases in the field of jujube tree using multi-omics combined with GWAS, and a standardized candidate gene mining process has not yet been formed.

The third-generation SNP/GBS markers also have knowledge gaps. Jujube tree populations generally have strong population stratification, and improper correction can lead to a large number of false positive associations. Due to the insufficient number of available large segregating populations, most of the associated loci obtained from GWAS are difficult to further transform into stable QTLs, and the interference of environmental interactions on locus effects has not been fully analyzed.

#### 3.2.3. Research on Domestication and Evolution of Jujube

Third-generation molecular markers have advanced jujube domestication and evolution research from traditional molecular markers to the genomic level, providing precise and comprehensive genomic evidence for analyzing the domestication origin and evolutionary pathways of jujube. Multiple studies based on SNPs and other markers [53,54,55,56] have confirmed that wild jujube is the ancestral species of cultivated jujube, and that the domestication from wild jujube to cultivated jujube was accomplished through multiple pathways. Yang Meng et al. [1] analyzed the chloroplast genomes of 326 jujube plants (133 cultivated jujubes and 193 wild jujubes), revealing two domestication pathways for cultivated jujubes: one driven by natural selection with less human interference, and the other dominated by human domestication and cultivation. This study provided a new genomic perspective for research on jujube domestication and evolution, enriching the findings on the origin and evolution of jujube trees.

Meanwhile, SNP markers have also been applied to studies of genetic diversity and genetic differentiation in wild jujubes. Shao Lingzhi et al. [57] employed Indel’s species identification markers to conduct a systematic analysis and identification of the genetic relationship between wild jujube (Ziziphus acidojujuba) and cultivated jujube (Ziziphus jujuba). Noticeable alterations were detected under appropriate climatic conditions, and it was determined that the average temperature in the coldest season was the primary factor influencing the distribution. Consequently, considering the distinct suitable habitats, diverse protection strategies are required. For instance, cultivated jujubes have a wider distribution, while wild jujubes are concentrated in northern China. Zhang Dapeng et al. [58] analyzed 177 American jujube accessions using 147 SNP markers, detecting 74 unique genotypes and revealing the genetic diversity and population structure of American jujube resources. Nisar Uddin et al. [59] used GBS technology to analyze 200 genotypes from five jujube species originating from China and Pakistan, identifying 10,945 high-quality SNP loci and discovering that wild jujube plants in Pakistan possess richer genetic diversity. These findings provide a reference for the genetic improvement and domestication evolution research of jujube plants. The current research only focuses on SNP single-base variations, and key domestication variations such as PAV, CNV, and large fragment SV discovered through pan genome mining have not been systematically developed as molecular markers.

### 3.3. A Comparative Study on the Application Effects of Different Molecular Marker Technologies in Jujube Research

In the research of jujube trees, second-generation and third-generation molecular markers each possess distinct advantages and limitations. Noticeable disparities exist among them in terms of application scenarios, research depth, and technical requirements. The comparison of their application effects is presented in Table 3. In practical research, these markers can be applied in a collaborative manner, leveraging each other’s strengths in accordance with the research objectives and conditions.

Overall, among second-generation molecular markers, SSR markers currently serve as the “basic core markers” in jujube research. Due to their strong stability and rich polymorphism, they remain widely used in fundamental studies such as germplasm resource identification and genetic diversity analysis in jujube. In contrast, SNP and InDel markers, as third-generation molecular markers, represent the “developmental core markers” in jujube research. They meet the demands of in-depth genetic basis analysis and molecular breeding research, and have become the mainstream direction in jujube molecular marker studies. These two types of markers are not in a substitutive relationship but rather a complementary and collaborative one. In practical research, they can be used in combination; for example, SSR markers can be employed for initial germplasm resource identification, followed by SNP markers for subsequent high-density gene mapping and genetic analysis, thereby improving research efficiency and depth.

## 4. Problems and Challenges in Molecular Marker Research of Jujube Trees

Molecular marker technology has become a core technical tool in the research fields of jujube genetic breeding, germplasm resource evaluation, and genomic research. Multiple molecular marker techniques, including RAPD, AFLP, SSR, SNP, and RAD, have been successively applied to jujube-related research, which has produced remarkable research results and promoted the rapid development of molecular genetics research on jujube. However, current research on jujube molecular markers still faces many key challenges, which hinder the efficient transformation and application of molecular marker technology in jujube breeding practice. These challenges are mainly reflected in the following four aspects.

### 4.1. Inherent Defects in Molecular Marker Development and Sequencing Technology

First, the development of functional molecular markers is severely insufficient. Most of the molecular markers currently applied are neutral markers that are not associated with phenotypic traits, while the development of functional markers closely linked to key target traits such as disease resistance, high quality, high yield, and stress tolerance lags significantly. Even the few developed functional markers have weak trait association effects, leading to low prediction accuracy, which is far from meeting the practical requirements of jujube precision breeding and molecular design breeding. This defect has become a core problem restricting the efficient application of molecular marker technology in jujube breeding. Second, a large-scale molecular marker detection system for jujube based on a high-quality T2T haplotype genome has not yet been established. High-throughput SNP chip development and KASP marker development are still in a blank state, and there is a lack of standardized high-throughput molecular marker detection platforms. Third, the inherent errors of high-throughput sequencing technology interfere with the reliability of markers: ① There are assembly gaps in the local repetitive regions of the T2T genome, and SNP alignment produces false positive variations. ② GBS low coverage sequencing results in a large number of missing genotypes, leading to deviations in LD calculation and GWAS localization. ③ PCR amplification preference and uneven restriction enzyme efficiency introduce typing errors. ④ The triploid jujube germplasm lacks suitable SNP typing algorithms, resulting in low accuracy in identifying heterozygous loci. Fourthly, existing markers only focus on single-nucleotide SNPs and short-fragment InDel, and pan-genomic analysis of PAV, CNV, and large-fragment structural variations has not been transformed into molecular markers that can be used for breeding, resulting in the loss of a significant amount of key trait regulatory variation resources.

### 4.2. The Disconnect Between Molecular Markers and Breeding Practice Transformation

The application of developed molecular markers in jujube breeding practice is still insufficient, and the genome-assisted selection breeding system has not been fully established. Most current molecular marker research still stays at the basic research level, and molecular marker technology has not been organically integrated with traditional jujube breeding techniques [60]. The technical advantages of molecular markers have not been effectively transformed into actual breeding outputs, which makes jujube breeding still rely on traditional phenotypic selection, and the overall breeding efficiency needs to be further improved.

### 4.3. Data Silos and Lack of Standardization System

The molecular markers developed by different research teams currently lack a unified database management system, leading to a prominent “data silo” issue. Marker sequences, genotyping protocols, and research results from independent studies are difficult to effectively integrate and compare across different research projects. This situation not only causes repetitive waste of marker resources and unnecessary consumption of research costs, but also severely hinders collaborative progress and knowledge sharing in jujube molecular marker research, and greatly impedes the standardization and large-scale development of jujube molecular marker technology.

### 4.4. Insufficient Integration of Emerging Multi-Omics and Cross-Disciplinary Technologies

The application of emerging technologies including gene editing, pan-genomics, artificial intelligence, and single-cell sequencing in the development of jujube molecular markers remains insufficient. These technologies have not fully exploited the unique advantages of artificial intelligence in optimizing the development process of molecular markers and analyzing the genetic basis of complex traits. Additionally, they have not effectively integrated the synergistic effects of gene editing in functional gene validation, pan-genomics in comprehensive analysis of genetic variation, and single-cell sequencing in the development of tissue-specific markers. These multi-dimensional constraints limit the innovative development and research depth of jujube molecular marker technology, and further restrict the potential of this field to advance to a higher level.

## 5. Future Development Directions and Countermeasures of Molecular Marker Research on Jujube Trees

Against the backdrop of the current problems in jujube molecular marker research and the development trend of plant molecular breeding at home and abroad, future research should focus on core directions including functional marker development, high-throughput detection system construction, breeding application transformation, and integration of emerging technologies.

### 5.1. Functional Marker Development and High-Throughput Genotyping Platforms

Recent advances in whole-genome resequencing have substantially expanded the marker resources available for jujube. For instance, analysis of resequencing data from 460 representative jujube accessions identified 585,131 high-quality SNPs, from which 100 core KASP markers were developed, with an average polymorphism information content (PIC) of 0.412 and a Shannon–Weiner index of 0.868, indicating high informativeness and discriminatory power. A core collection of 92 accessions was subsequently established, and a DNA fingerprinting system covering all 460 germplasms was constructed. Of the 100 candidate markers, 23 SNPs were successfully converted into functional KASP markers (conversion rate 57.5%), and these markers demonstrated robust discriminatory power in distinguishing 46 natural population samples and 50 hybrid progenies [61].

Moreover, a recent patent report disclosed 100 high-quality SNP markers developed for jujube germplasm identification, with 23 successfully converted to KASP markers for genetic diversity analysis and cultivar authentication [62]. Another study reported the development of a SNP marker located in the promoter region of the *ZjAC03* gene (at position –484A>G), which is significantly associated with citrate content in jujube fruits, and validated its utility via KASP genotyping for early selection of low-acid genotypes [63]. These examples demonstrate that functional marker development in jujube is transitioning from theoretical possibility to practical implementation. Leveraging the high-quality T2T haploid genome of jujube, future research should accelerate the development of haplotype-based molecular markers and standardized SNP chips. On the basis of the established 100-core KASP marker panel and the validated conversion protocols, we propose the following specific, quantitatively defined objectives.

SNP chip development: Develop a universal SNP typing chip for jujube containing 3000–5000 uniformly distributed functional loci covering all 12 linkage groups. The feasibility of this target is supported by the existing 585,131 high-quality SNPs identified from 460 accessions [61], which provide a sufficiently large pool for marker selection after stringent quality filtering (MAF ≥ 0.05, call rate ≥ 95%). KASP marker expansion: Screen ≥ 200 KASP functional markers associated with fruit quality, drought resistance, and disease resistance, building upon the 23 successfully validated markers [61] and the citrate-associated marker developed for *ZjAC03* [63]. Cost and throughput optimization: Achieve cost control within 100 RMB per sample for single-sample genotyping and enable batch testing of 384 germplasm samples per run, drawing on the established cost-effective nano-fluidic SNP genotyping protocols that can handle hundreds to thousands of samples per day [64]. Standardized development pipeline: Establish a standardized workflow of “pan-genomic variation mining → RNA-seq/eQTL screening of candidate genes → independent population marker validation” to enhance the predictive ability of functional markers for phenotypic traits.

### 5.2. Construction of a Whole-Genome Assisted Selection Breeding System

In the construction of a whole-genome-assisted selection breeding system for jujube trees. Taking molecular marker technology as the core and combining the achievements of functional gene mining, a jujube genome-wide-assisted selection breeding system integrating germplasm identification, trait localization, and molecular selection should be constructed to realize high-throughput and precise selection of important jujube agronomic traits.

It is worth noting that although marker-assisted selection and marker-assisted backcrossing have achieved preliminary applications in several jujube breeding programs for target traits such as fruit size and disease resistance, Hou Lu et al. [65] used GWAS to locate SNPs related to fruit size and cracking, and clearly proposed “Upon further validation, the significant markers identified in the present study may be useful for marker-assisted breeding to improve jujube fruit quality.” However, genomic selection and genomic prediction are still largely confined to basic research. The major bottlenecks include insufficient size of training populations and the absence of mature high-density marker panels for jujube. Therefore, future studies should prioritize the construction of large-scale reference populations with abundant and well-characterized phenotypic records, so as to promote the routine practical deployment of genomic selection in jujube breeding. Strengthen the organic integration of molecular marker technology and conventional jujube breeding technology, and incorporate molecular genotyping into the whole breeding workflow including germplasm screening, hybrid combination design, and progeny early-stage selection. For major-effect qualitative traits, MAS can be adopted to rapidly screen elite individuals. For cultivar improvement, MABC can be used to rapidly recover the genetic background of recurrent parents. For complex multi-gene traits such as fruit quality and stress resistance, a genome selection breeding value prediction model should be constructed based on existing 460 germplasm resequencing data. Strengthen the organic integration of molecular marker technology and conventional jujube tree breeding technology, promote the transformation of molecular marker technology from basic laboratory research to practical breeding applications, and shorten the breeding cycle of excellent new varieties.

### 5.3. Improvement of Molecular Marker Resource Sharing and Technical Standards

With respect to improving the molecular marker resource sharing mechanism and formulating relevant technical standards, it is necessary to strengthen cooperation and academic communication among domestic and international research teams and institutions, construct a unified database for jujube molecular markers, realize the sharing and integrated utilization of marker sequences, detection protocols and research data, break down existing “data silos”, improve the utilization efficiency of marker resources, and avoid redundant research. Furthermore, national and even international technical specifications for the development, verification, detection, and application of jujube molecular markers should be established to standardize the research and application of jujube molecular markers, ensure the consistency and comparability of data generated by different platforms and laboratories, and drive the standardized and large-scale development of jujube molecular marker technology.

The recently published core collection and DNA fingerprinting system covering 460 jujube germplasms provide a foundational resource for such standardization efforts. The QR-code-based identification system developed for representative varieties offers a practical model for resolving intellectual property disputes caused by homonymous varieties or varieties with different names but identical genotypes.

### 5.4. Integration of Emerging Technologies

The convergence of molecular marker research with emerging biotechnologies presents transformative opportunities: utilizing artificial intelligence and deep learning to optimize GWAS statistical models, correcting population structure, reducing false positives, and analyzing complex traits interacting in multiple environments. Using CRISPR/Cas9 [66] gene editing to verify the true function of candidate genes and provide precise targets for the development of functional markers. Utilizing pan-genomic systems to mine PAV, CNV, and large segment SV, and developing novel structural variation molecular markers. Using single-cell sequencing to mine tissue-specific variation markers in fruit pulp, leaves, and flowers, and improve organ-specific quality traits.

### 5.5. Integration of Industry–University–Research Collaboration and Patent Protection

In the aspect of strengthening industry–university–research integration and patent protection, it is necessary to promote deep industry–university–research collaboration among universities, research institutes, and breeding enterprises, jointly establish jujube molecular marker breeding centers, build transformation platforms connecting molecular marker basic research and breeding application, promote the industrial transformation and application of functional molecular markers, and improve the patent protection mechanism for molecular marker technology. Strengthening patent protection for original, low-cost, and high-value molecular markers can encourage researchers to carry out innovative molecular marker development research, promote the technological innovation and industrial development of jujube molecular markers, and provide solid technical support for the high-quality development of the jujube industry.

### 5.6. Systematic and Collaborative Application of Molecular Marker Technology

To promote the systematic and collaborative application of molecular marker technology, the traditional single-marker application mode should be transformed to the combined application of multiple markers. For example, the SSR+SNP marker combination is adopted for germplasm resource evaluation, while the SNP+InDel marker combination is applied for gene localization. This combinatorial application strategy effectively improves the application performance of molecular marker technology in germplasm resource evaluation, genetic analysis, breeding practice, and other research scenarios. Meanwhile, the integrated application of molecular markers and gene editing should be further explored. Molecular markers are first used for trait association analysis and target gene localization, then gene editing is employed for precise modification of target genes, and finally molecular markers are reused to verify the editing effect, so as to realize precise molecular design breeding of jujube. This approach can promote the leap of jujube breeding from traditional phenotypic selection to precise molecular design breeding.

## 6. Summary and Outlook

This paper systematically reviews the multi-dimensional classification system of plant molecular markers and the characteristics and selection criteria of common marker technologies. It is found that molecular marker research on jujube started relatively late but has developed rapidly. There are no reports on the application of first-generation molecular markers in jujube. Second-generation molecular markers marked the starting point of this research, among which SSR markers—owing to their strong stability and rich polymorphism—remain the core markers for basic research, such as germplasm resource identification and genetic diversity analysis. Third-generation molecular markers, centered on SNP and InDel, are gradually becoming the mainstream in jujube research, playing a key role in advanced studies, including high-density genetic mapping, important trait gene mapping, and domestication and evolution genomic analysis. These two types of markers exhibit a complementary and collaborative application pattern in jujube research.

Molecular marker technology has achieved remarkable results in the precise identification of jujube germplasm resources, genetic diversity analysis, genetic relationship determination, high-density genetic mapping, functional gene mapping, and domestication and evolution tracing. The publication of the high-quality T2T haplotype genome of jujube has also laid a solid genomic foundation for the development and application of molecular markers. However, current jujube molecular marker research still faces several challenges, including insufficient development of functional markers, a lack of high-throughput detection systems, inadequate integration of technology with breeding practices, an incomplete data sharing mechanism, and insufficient integration of emerging technologies. These issues restrict the efficient application of molecular marker technology.

Despite substantial progress in jujube molecular marker research, several fundamental breeding mysteries remain unresolved, including the genetic architecture of fruit shape and cracking, the regulatory networks controlling sugar accumulation, and the full spectrum of genomic variation underlying domestication. To address these, future studies should expand GWAS panels with wild germplasm, integrate multi-omics (transcriptomics, metabolomics, proteomics) to decipher regulatory cascades, and construct graph-based pan-genomes to capture structural variations (CNVs, PAVs) that are invisible to SNP-only approaches. Fine-mapping and functional validation using CRISPR/Cas9 will be essential to pinpoint causal genes, such as those recently identified for fruit cracking [65] (PMAT1, WRKY41) and sugar transport [67] (the *ZjABF1-ZjSWEET* module). These efforts will enable marker-assisted selection for complex traits while revealing how structural variants contribute to phenotypic diversity and stress resilience.

Equally promising are emerging technologies beyond conventional markers. CRISPR/Cas9 not only enables functional validation of candidate genes but also offers a path toward precise gene editing for quality improvement and can generate CRISPR-derived diagnostic markers. Epigenetic regulation—particularly DNA methylation—has been shown to affect fruit development and domestication in jujube [68], and future epigenome-wide association studies (EWAS) could identify methylation QTLs linked to agronomic traits. The convergence of pan-genomics, gene editing, and epigenetics, together with high-throughput phenotyping and computational modeling, holds the potential to realize molecular-design breeding. However, this vision requires sustained investment in germplasm characterization, transformation system optimization, and interdisciplinary collaboration. Ultimately, the lingering mysteries are not barriers but signposts guiding the next frontier of jujube genetics and breeding.

In the future, jujube molecular marker research should focus on the development of functional markers and the construction of high-throughput detection systems, aim to establish a comprehensive genome-wide assisted selection breeding system, be driven by emerging technologies, strengthen industry–academia–research integration, improve data sharing mechanisms and technical standards, and promote the systematic and collaborative application of molecular marker technology. The combined application of SSR and SNP markers will become a trend. By integrating multi-omics, gene editing, artificial intelligence, pan-genomics, and other emerging technologies, future research will further promote innovation and development in jujube molecular marker technology, accelerate the establishment of a molecular design breeding system for jujube, and achieve a leap from traditional phenotypic selection to precise molecular design breeding in jujube. This will provide strong technical support for the selection of new jujube varieties with high yield, high quality, and strong adaptability, and promote the high-quality development of China’s jujube industry.

## Figures and Tables

**Table 1:** Category, advantages, and limitations of function-related molecular markers.

Marker/Assay	Category	Advantages	Limitations
Anonymous SSRs	Neutral marker	Abundant genomic distribution High polymorphism Well-established protocols	Largely unlinked to functional genes Poor transferability across germplasm
Gene-derived SSRs	Putative functional marker	Anchored to genic regions Potential trait-linkage	Limited number Low polymorphism within conserved genes
EST-SSRs	Putative functional marker	Derived from transcribed regions Favorable cross-germplasm transferability	Restricted to coding regions Low genome-wide coverage
CAPS	Putative functional marker	Co-dominant inheritance Low equipment requirement Genic anchoring	Dependent on native restriction-enzyme sites Few available loci Low throughput
dCAPS	Putative functional marker	Bypasses requirement for native restriction sites; Co-dominant inheritance	Challenging primer design Unstable amplification Low throughput
Functional SNPs	Functional marker	May represent causal variants Abundant across the genome	Require experimental validation Rely on high-throughput genotyping infrastructure
KASP	Genotyping platform (not an independent marker type)	High-throughput and automated Suitable for large-population validation	Serves only as an assay Prior knowledge of SNP sequences is mandatory

**Table 2:** Commonly used molecular markers and their characteristics.

Type	Genetic Property	Polymorphism	Repeatability	Prior Sequence Information	Technical Complexity	Throughput	Cost
RFLP	Codominant	Medium	High	Not Required	High	Low	High
RAPD	Dominant	Medium	Low	Not Required	Low	Low	Low
AFLP	Codominant or Dominant	High	Medium-high	Not Required	High	Medium-high	Medium-high
SSR	Codominant	High	High	Required	Medium	Medium-low	Medium
ISSR	Dominant	High	Medium	Not Required	Medium	Medium	Low
SNP	Codominant	Medium-high	High	Required	High	High	Low
KASP	Codominant	Medium	High	Required	High	Medium	Medium

**Table 3:** Comparison of the application effects of different molecular marker techniques in jujube trees.

Molecular Marker Generation	Core Marker Type	Technical Advantages	Limitations	Application Scenarios	Research Depth
Second generation	SSR AFLP ISSR RAPD	Simple operation Moderate cost No high-throughput sequencing platform required Good stability	Low throughput Limited genomic coverage Difficult to satisfy large-scale research requirements	Genetic resource identification Genetic diversity analysis Clonal relationship determination Early genetic map construction	At the fundamental level Mainly for germplasm resource evaluation
Third generation	SNP InDel RAD GBS	High throughput High accuracy Wide genomic coverage Capable of large-scale genotyping	High initial platform investment Requirement for bioinformatic analysis capacity High-quality DNA required	Construction of high-density genetic map Mapping of important trait-related genes GWAS Genomic analysis of domestication and evolution	At a deeper research level Focuses on genetic-basis dissection and functional gene mining

## Data Availability

The original contributions presented in this study are included in the article. Further inquiries can be directed to the corresponding author.
